# Radiation mitigating activities of *Psidium guajava* L. against whole-body X-ray-induced damages in albino Wistar rat model

**DOI:** 10.1007/s13205-020-02484-y

**Published:** 2020-11-04

**Authors:** Amith Kumar, Reshma Kumarchandra, Rajalakshmi Rai, Vasavi Kumblekar

**Affiliations:** 1 Department of Biochemistry, Kasturba Medical College, Mangalore, Manipal Academy of Higher Education, Manipal, Karnataka India; 2 Department of Anatomy, Kasturba Medical College, Mangalore, Manipal Academy of Higher Education, Manipal, Karnataka India

**Keywords:** Antioxidant activity, Flavonoids, Genotoxicity, Inflammatory markers, Immunosupression

## Abstract

In the present study, we investigated radiation mitigating activities of *Psidium guajava* L. (*P. guajava*) against whole-body X- ray induced damages in albino Wistar rat model. The animals were orally administered with 200 mg/kg bodyweight of hydroalcoholic leaf extract of *P. guajava* for five consecutive days and on the fifth day, after the last dose of extract administration, animals were exposed to 4 Gy of X-rays. Rats were sacrificed 24 h post X–ray irradiation. The radiomitigating activity of the herb extract was assessed by micronucleus assay, histopathology of the small intestine and hematological parameters. Hepatic cyclooxygenase–2 (COX-2), interleukin–6 (IL-6) and interleukin –10 (IL-10) levels were assayed to validate the anti-inflammatory property. Biochemical estimations were also performed in RBC lysates to corroborate antioxidant properties in the leaf extract. HPLC analysis of crude extract confirmed the presence of standard flavonoid quercetin. Our results indicated that radiation elevated COX-2, IL-6 and decreased IL-10 levels and also induced micronucleus formation in polychromatic erythrocytes, simultaneously impairing hematological parameters along with erythrocyte antioxidants. The animals pre-treated with *P. guajava* exhibited a significant decrease in the COX-2 (*P* ≤ 0.01), IL-6 levels (*P* ≤ 0.05) and also displayed significant increase in the hepatic IL-10 levels (*P* ≤ 0.01). Pre-treatment with plant extract improved antioxidant enzyme activities, hematological parameters and reduced the intestinal damage by recovering the architecture of the small intestine. Moreover, extract also rendered protection against radiation induced DNA damage, as evidenced by the significant (*P* ≤ 0.01) decrease in the percentage of radiation-induced micronucleus in polychromatic erythrocytes. Furthermore, the herb extract treatment increased radiation LD_50/30_ from 6.6 Gy to 9.0 Gy, offering a dose reduction factor (DRF) of 1.36. Our findings for the first time propose the beneficial use of *P. guajava* as a radioprotector against X-ray induced damage.

## Introduction

Usage of nuclear power and radioactive nuclide by humans, as in agriculture, nuclear medicine, food preservation, power generation, industries and military applications increases the risk of exposure to lethal ionizing radiation in all living beings (Cuttler et al. 2009). In this context, safeguarding humans from the deleterious effect of radiation is a primary challenge. Exposure of radiation to biological system triggers the production of reactive oxygen species (ROS) and reactive nitrogen species (RNS) depleting the endogenous antioxidant status of a cell. ROS also reacts with vital macromolecules such as DNA and membrane lipids leading to altered gene expression and loss of cellular osmotic balance resulting in cell death (Borek 2004). Acute whole-body exposure of ionizing radiation damages actively proliferating cells present in bone marrow, gastrointestinal tract and central nervous system (Akeem et al. 2019). A good level of antioxidants in the body during exposure to ionizing radiation reduces the genotoxicity induced by radiation (Weiss et al. 2003). Numerous chemical constituents have been tested to date for their ability to guard against radiation induced cellular damage. However, this practice is constrained due to toxicity, high cost and their side effects (Rosen et al. 2015). In the prevailing scenario, efforts are being made to develop effective, nontoxic drugs against radiation induced damage, originating from herbs. Natural products are used as radioprotectors in recent years due to their appreciable antioxidant activity, reduced toxicity, easy availability and their pharmacological benefits (Hosseinimehr 2007). *Psidium guajava* L. commonly called guava belongs to the family Myrtaceae and is widely cultivated in tropical and subtropical regions around the world. It is an evergreen shrub native to South America, Mexico, Asia and Central America (Diaz-de-Cerio et al. 2017). The leaves of *P. guajava* are used traditionally to treat a plethora of human ailments which includes hypertension, gastroenteritis, diarrhea, diabetes mellitus, antispasmodic, ulcers, inflammatory conditions, anorexia, cholera, skin problems, bacterial infections, toothaches, constipation and itchy rashes caused by scabies (Gutierrez et al. 2008). Presence of lycopene content in guava blocks the oxidative damage of nucleic acids, proteins and lipids generated by free radicals (Basu et al. 2007). Aqueous leaf extract of *P. guajava* being a potential source of natural antioxidants, protected human dermal fibroblast cells against cytotoxic damages initiated by reactive oxygen species (Alvarez-Suarez et al. 2018). Hydroalcoholic leaf extract exhibited effective anti-inflammatory and analgesic properties in rats (Jung et al. 2014). The aqueous leaf extract strongly suppressed inflammation and oxidative stress in rats treated with streptozotocin (Li et al. 2015). Considering the profound medicinal property of *P. guajava*, the present work aims at studying the radiation mitigating activity of this plant, in albino Wistar rats exposed to whole-body X-rays, which has not been reported to date.

## Materials and methods

### Chemicals

Bovine serum albumin (BSA), Giemsa, 2,2-Diphenyl-1-picrylhydrazyl (DPPH), sodium nitroprusside, quercetin, sulphanilamide, phosphoric acid, tri sodium citrate, ammonium molybdate, sodium chloride, hydrogen peroxide_,_ thiobarbituric acid (TBA), trichloroacetic acid (TCA), hydrochloric acid, 2,4-Dinitrophenylhydrazine (DNPH), malonaldehyde-bis-dimethyl acetal (MDA) and May-Grunwald’s stain were procured from Sigma-Aldrich ( St Louis, USA). Rat COX-2 ELISA kit was obtained from Genxbio health sciences Pvt Ltd, India. Rat IL-6 ELISA kit and Rat IL-10 ELISA kit was procured from Ray Biotech Pvt Ltd, India. All other chemicals and reagents used were of analytical grade.

### Radiation source

**Fig. 1:**
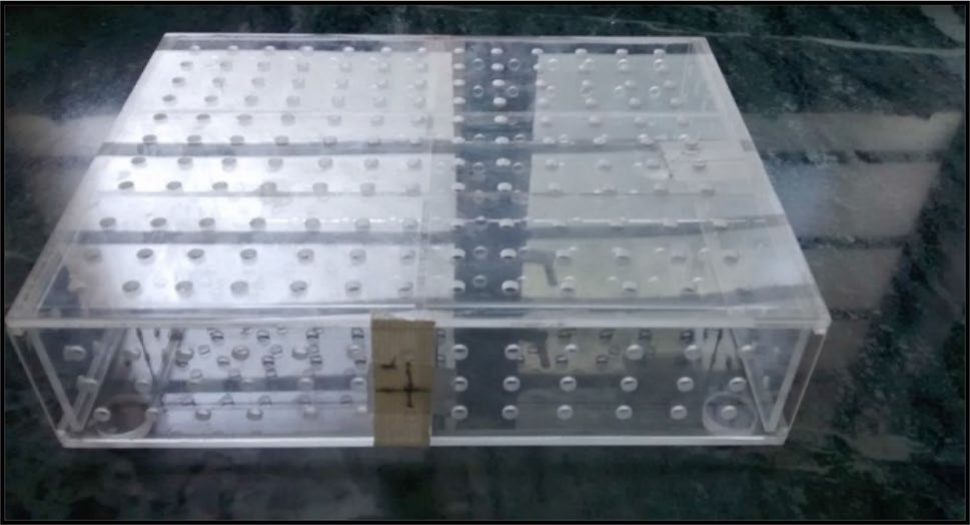
Perspex box restrainer

**Fig. 2:**
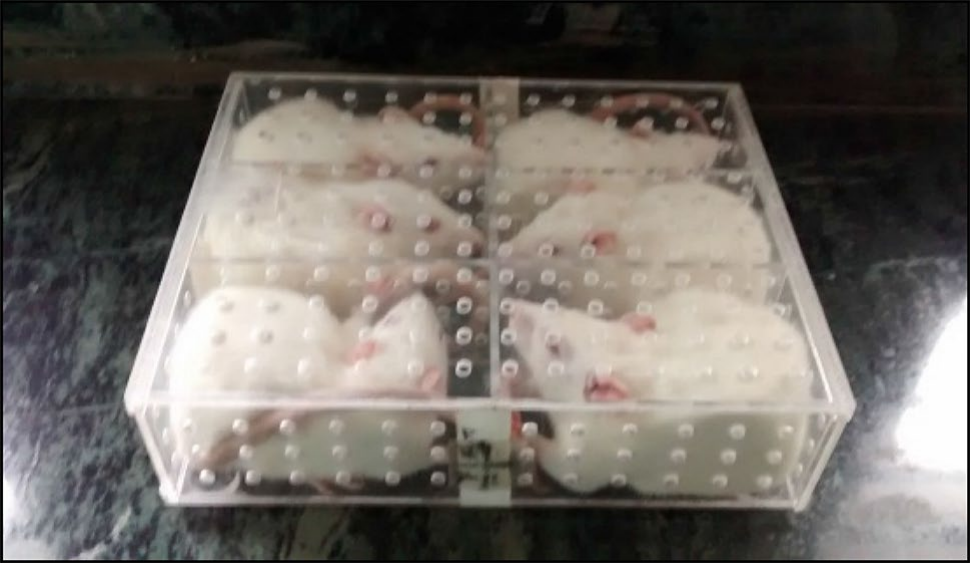
Animals restrained in a perspex box restrainer

**Fig. 3:**
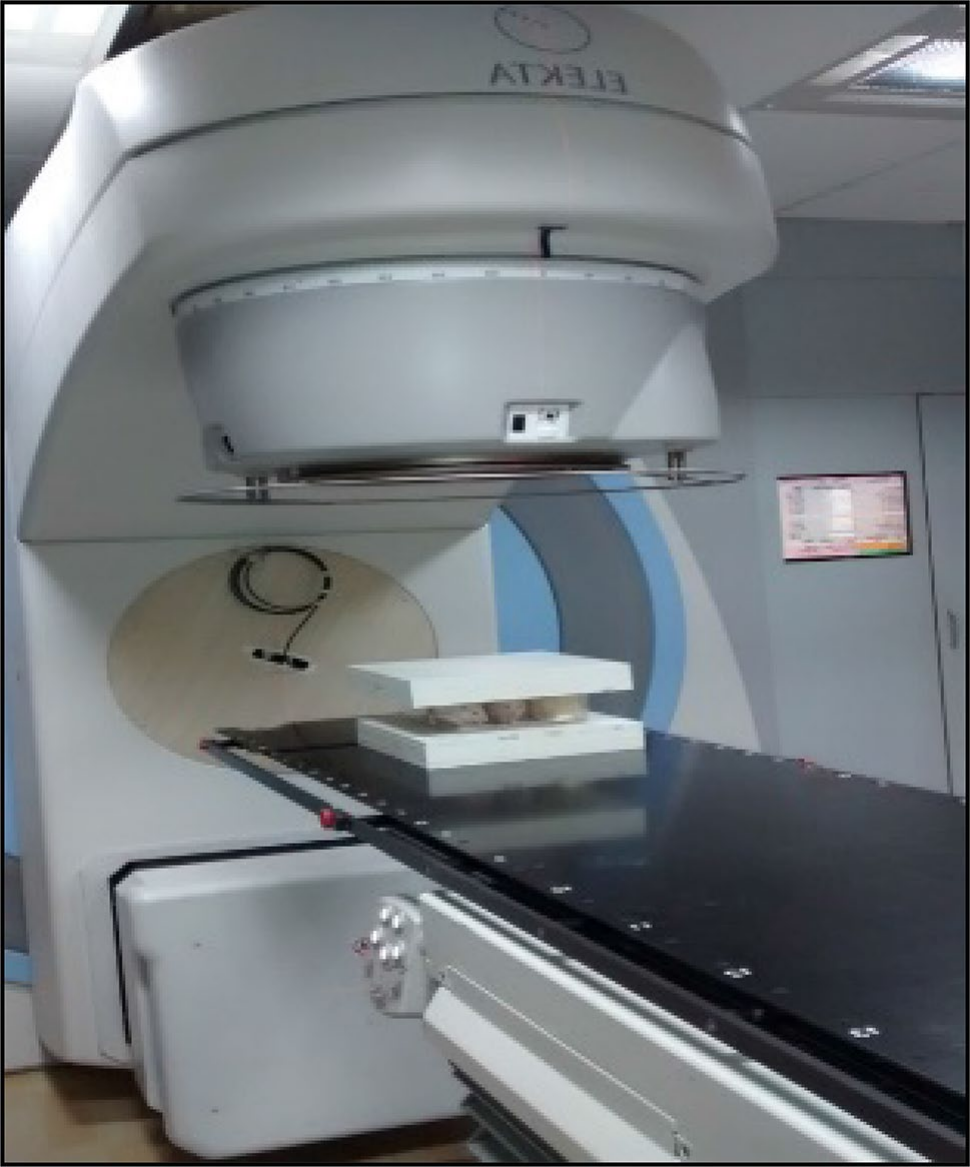
Procedural description of LINAC (Linear accelerator) whole-body X-ray exposure

All animals were placed in a rectangular box restrainer made of perspex material (Figs. 1 and 2). The box was divided into two compartments, and three rats were placed in each compartment (18 cm × 18 cm width and 22.2 cm length, partitioned into 6 cm × 6 cm × 11.1 cm). The source of radiation was LINAC accelerator, (Linear accelerator—Fig. 3), Department of Radiotherapy, Kasturba Medical College Hospital, Attavar, Mangalore, India. Totally six animals were whole- body irradiated with X-rays using a dose rate of 3.5 Gy/minutes. The distance from the beam exit point to animals was 100 cm. The field size of LINAC accelerator was 40 × 40 cm^2^.

### Animals care and handling

The present study was carried out in albino Wistar rats of either sex, weighing 160 ± 10 g. The animals used in this experiment were 8–10 weeks. The present study was approved by the Institutional Animal Ethics Committee (IAEC) Manipal Academy of Higher Education. The animals were obtained from Central animal house, Kasturba Medical College, Bejai, Mangalore (Reg.No.213/CPCSEA).

### Plant material and extraction

The tender leaves of *P.guajava* were collected from Soans nursery, Alangar, Moodbidri, Mangalore India. The plant material was authenticated by botanist Surendranath Joshi from St. Aloysius College, Mangalore. These leaves were washed properly in running tap water and shade dried for about 15 days. After proper shade drying of leaves, the material was blended in an electric mixer to get a fine powder. About 100 g of dried plant material was suspended in a thimble in the extractor of Soxhlet apparatus. The round bottom flask consisted of 500 ml of 50% methanol and water which was refluxed for 72 h at 50 °C (Poojary et al. 2019). The resulting crude extract was concentrated by a rotary vacuum flash evaporator and stored at 4 °C for further use.

## In vitro studies

### 2-Diphenyl-1-picrylhydrazyl (DPPH) scavenging activity

DPPH free radical scavenging activity of *P. guajava* was determined by the modified method described by (Liu et al. 2008). Aliquots containing various concentrations of *P. guajava* (2–10 µg/ml) in a final volume of 1 ml were mixed with 1 ml of 0.05 mM DPPH made in methanol. An equal amount of methanol served as control. Quercetin (2–10 µg/ml) was used as a standard for comparison. The samples were incubated for 20 min at 37 °C and the optical density was observed at 517 nm. Analysis was done in triplicates and percentage scavenging was calculated using the formula.

$$ {\text{Percentage scavenging }}\left( \% \right) \, = \, \left[ {\left( {{\text{A}}_{0} - {\text{A}}_{{1}} } \right)/{\text{A}}_{0} \times { 1}00} \right] $$

(A_1_ is absorbance of standard/test, A_0_ is absorbance of control).

### Nitric oxide scavenging activity

The nitric oxide scavenging activity of *P. guajava* was measured spectrophotometrically using Griess reagent as described by Jagetia and Baliga (2004). At physiological pH, sodium nitroprusside spontaneously produces nitric oxide which acts with surrounding oxygen to produce nitrite ions which react with Griess reagent developing pink colour. 0.5 ml of different concentrations of plant extract and standards were mixed with 2 ml of 10 mM sodium nitroprusside. To this reaction mixture 0.5ml of Phosphate buffer saline pH 7.4 was added and incubated at 25 °C for 150 min. To 0.5 ml of the above solution 0.5 ml of Griess reagent (1% sulphanilamide, 2% H_3_PO_4_ and 0.1% napthylethylene diamine dihydrochloride) was added. The nitrite present in a sample reacts with sulphanilamide in a subsequent coupling with napthylethylene diamine dihydrochloride to give a chromophore which is read at 546 nm. Ascorbic acid was used as a standard for comparison. The percentage scavenging activity of nitric oxide in samples as well as in standards were calculated by the following formula.

$$ {\text{Percentage scavenging }}\left( \% \right) \, = \, \left[ {\left( {{\text{A}}_{0} - {\text{A}}_{{1}} } \right)/{\text{A}}_{0} \times { 1}00} \right] $$

(A_1_ is absorbance of standard/test, A_0_ is absorbance of control).

### HPLC analysis of *P. guajava*

Hydroalcoholic leaf extract of *P. guajava* was evaluated for the presence of standard flavonoid quercetin by HPLC method. Alliance 2690 separation module (Dual Lambda Absorbance Detection) made by Waters Pvt Ltd was used. The system was maintained by Empower II software. The analysis was carried out by the column, Varian Microsorb C8 of diameter 250 × 4.6 with 5 µm particle size. 10 mg in 1 ml of standard quercetin was prepared in methanol. The set run time was 30 min. The flow rate was 1 ml/min. The flavonoid content was identified depending on the retention time of standards.

### Experimental design

To study the radiation mitigating property of *P. guajava*, the experimental animals were divided into four groups.

Group 1 (DDW) – All animals were treated with 1 ml/kg body weight of double-distilled water.

Group 2 (*P.guajava*) – All animals were orally administered with 200 mg/kg bodyweight of *P. guajava* for five consecutive days.

Group 3 (DDW + X-rays)—All animals were administered with 1 ml/kg body weight of double-distilled water and exposed to 4 Gy, sublethal dose of X-rays.

Group 4 (*P. guajava* + X-rays) – *P. guajava* (200 mg/kg bodyweight) was administered to experimental animals for five continuous days. On the fifth day after administration of the last dose, animals were exposed to 4 Gy of X- rays.

### In vivo studies

All animals were sacrificed by cervical dislocation, 24 h post exposure to the last dose of radiation. Blood was collected by cardiac puncture and stored in sterile EDTA vacutainers for hematological analysis. The livers were perfused with ice-cold saline. The liver homogenates were prepared by suspending 1-g liver tissue in 10 ml phosphate buffer saline of pH 7.4. The homogenates were centrifuged at 12,000 rpm for 20 min in a cooling centrifuge (Remi CM -12). The supernatant was used to analyze the levels of IL-6, IL-10 and COX-2. The antioxidant activity of *P. guajava* was analysed in RBC lysates by assessment of biochemical parameters such as catalase, superoxide dismutase, lipid peroxidation and protein carbonyls. The homogeniser used for the preparation of homogenates was RO-1727A Remi Motors. Homogenisation process was done in chilled condition. The femur and tibia bones were removed and bone marrow micronucleus assay was performed. The jejunum was excised from animals and subjected to histopathological analysis.

## Effect of X-rays on various inflammatory markers

### Determination of COX- 2 levels in liver tissue homogenates

Rat liver COX-2 was determined in liver tissue homogenates using specific enzyme-linked immunosorbent assay kits (Genxbio rat COX-2 ELISA). The results were expressed in ng/ml.

### Determination of IL-6 levels in liver tissue homogenates

IL-6 levels were studied in rat liver tissue homogenates by ELISA method. Rat IL-6 Ray biotech kits were used in the present study. The IL-6 levels were expressed in pg /ml.

### Determination of IL-10 levels in liver tissue homogenates

IL-10 levels were assessed in liver tissue homogenates by ELISA method. Rat IL-10 Ray biotech kits were used in the present study. The IL-10 levels were expressed in pg/ml.

### Micronucleus assay

The micronucleus assay was done according to the procedure described by Schmid (Schmid 1975). The femur and tibia bones were excised and bone marrow cells were flushed in 1 ml of 5% BSA. The suspension was mixed and centrifuged at 1000 rpm for 10 min. The supernatant was removed and the cell pellets were suspended in fresh 5% BSA. The cell suspension was smeared on a microscopic slide to form a uniform smear and air-dried thereafter. The samples were methanol fixed and stained with May-Grunwald’s stain for 3 min. The smear was stained with Giemsa for 10 min. The Giemsa stains nucleus which appears dark blue in colour. About 2000 cells were scored in each animal by an oil immersion microscope.

### Hematology

Blood was collected by cardiac puncture and stored in labeled sterile EDTA vacutainers. Red blood cells, white blood cells and platelet count were determined 24 h post X-rays irradiation by standard laboratory procedures.

### Histopathological studies

Intestinal tissues were fixed in 10% formalin solution and dehydrated in ethanol, dehydrated in xylene and embedded in paraffin wax. Sections of 5 μ thickness were made using a microtome, and stained with hematoxylin–eosin and observed under a microscope.

## Biochemical estimations in RBC lysates

### Catalase (CAT)

Catalase activity was measured by the method described by Beers and Sizer (1952). The catalase activity in the sample was measured by decomposition of hydrogen peroxide into water molecules with a reduction in absorbance at 240 nm.

### Superoxide dismutase (SOD)

The estimation of SOD activity was carried out by the method described by Beauchamp and Fridovich (1971). Nitroblue tetrazolium chloride reacts with superoxide anions developed upon illumination with riboflavin and methionine to produce formazan which is blue colored complex. The enzyme present in the sample is calculated using the formula U/mg protein.

### Lipid peroxidation (LPO)

The estimation of lipid peroxidation was carried out by the method described by Buege and Aust (1978). Break down of polyunsaturated fatty acids produces Malonaldehyde which is a convenient index to determine the extent of lipid peroxidation. Malonaldehyde reacts with TBA to give a pink colour complex. The results are expressed in μmole MDA/gram of Hb.

### Estimation of protein carbonyls

The protein carbonyl content in the sample was estimated by 2,4-Dinitrophenylhydrazine method described by Stadtman and Livene (2006). DNPH reacts with carbonyl groups present in proteins leading to the formation of 2,4 -dinitrophenylhydrazone which can be detected and quantified spectrophotometrically.

### Preparation of RBC lysates

The preparation of RBC lysates was done according to the method described by Varashree and Gopalkrishna (2010). The collected blood samples were centrifuged at 5000 rpm for 10 min. The plasma and buffy coat were discarded. The packed cells were lysed with 1.5 volumes of chilled water. The solution was centrifuged at 16,000 ×g for 20 min. The supernatant was used for biochemical estimations.

### Determination of dose reduction factor (DRF) of *P. guajava*

To determine dose reduction factor of *P.guajava*, animals were divided into six groups. 10 animals were maintained in each group. The first group of animals received double-distilled water and maintained as normal control. 2nd, 3rd, 4th, 5th and 6th groups received a single dose of 200 mg/kg body weight, *P. guajava* for five consecutive days. On the fifth day after administration of the last dose of extract, animals were exposed to different doses of X-rays such as 7 Gy, 8 Gy, 9 Gy, 10G and 11 Gy. Water and food were provided *ad libtum* to the animals. Radiation sickness and mortality were observed for 30 days.

$$ {\text{DRF}} = \frac{{{\text{Mean}}{\kern 1pt} {\kern 1pt} {\text{lethal}}{\kern 1pt} {\kern 1pt} {\text{dose}}{\kern 1pt} ({\text{LD}}_{50/30} ){\kern 1pt} {\kern 1pt} {\text{of}}{\kern 1pt} P.{\kern 1pt} guajava + {\text{X } - \text{ rays}}{\kern 1pt} {\kern 1pt} {\text{treated}}{\kern 1pt} {\text{group}}}}{{{\text{Mean}}{\kern 1pt} {\kern 1pt} {\text{lethal}}{\kern 1pt} {\kern 1pt} {\text{dose}}{\kern 1pt} ({\text{LD}}_{50/30} ){\kern 1pt} {\kern 1pt} {\text{of}}{\kern 1pt} {\kern 1pt} {\text{X } - \text{ rays}}{\kern 1pt} {\kern 1pt} {\text{treated}}{\kern 1pt} {\text{group}}}} $$

### Statistical analysis

All results were expressed as Mean ± SEM. Data were analysed by one-way analysis of variance (ANOVA) following post hoc test Tukey using IBM SPSS statistics 20. *P* < 0.05 was considered significant.

## Results

### DPPH Free radical scavenging activity

**Fig. 4:**
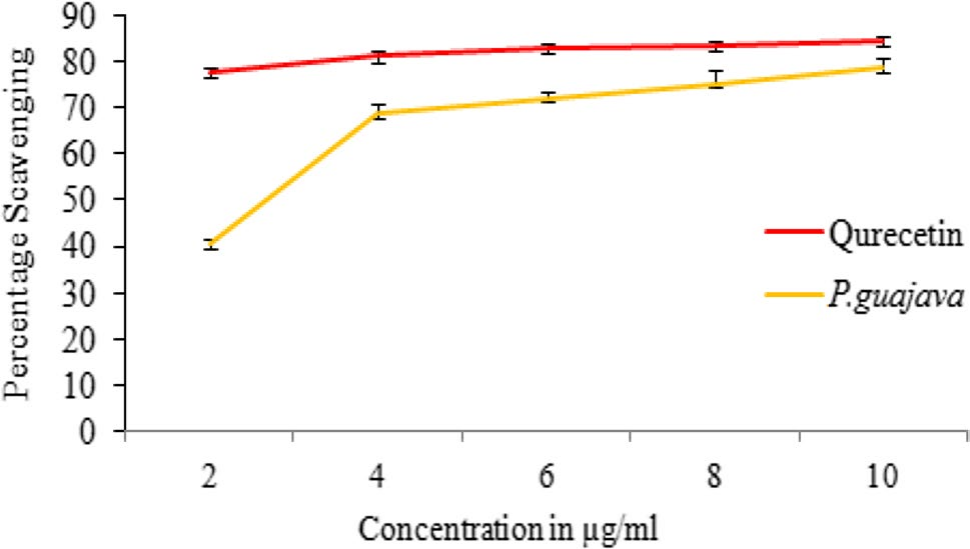
DPPH free radical scavenging activity of *P. guajava* at various concentrations

*P. guajava* extracts at different concentration in vitro displayed concentration dependent inhibition of DPPH free radicals. At a concentration of 10 µg/ml, 78.80% of DPPH free radical scavenging was observed which was maximum (Fig. 4).

### Nitric oxide scavenging activity

**Fig. 5:**
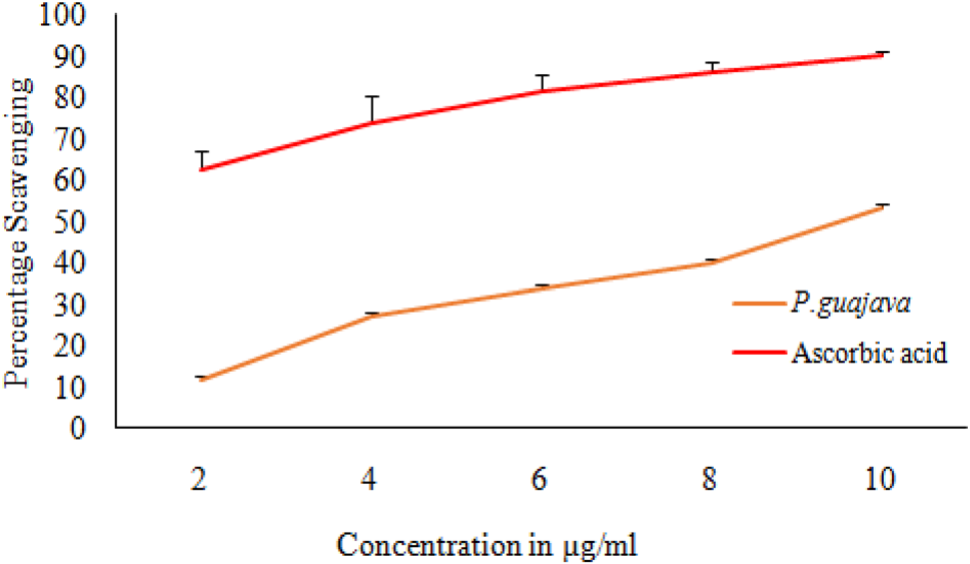
Nitric oxide scavenging activity of *P. guajava* at various concentration incubated with sodium nitroprusside solution

Incubation of sodium nitroprusside solution in phosphate buffer saline at 25 °C for 150 min with different concentrations of the extract, resulted in a linear time dependent nitrite production. The released nitrite was reduced by *P.guajava* in a concentration dependent manner. A maximum reduction of 53.16% nitric oxide radicals was observed at a concentration of 10 μg/ml (Fig. 5).

### Effect of *P. guajava* on radiation induced inflammation

#### Estimation of COX-2 in liver tissue

**Fig. 6:**
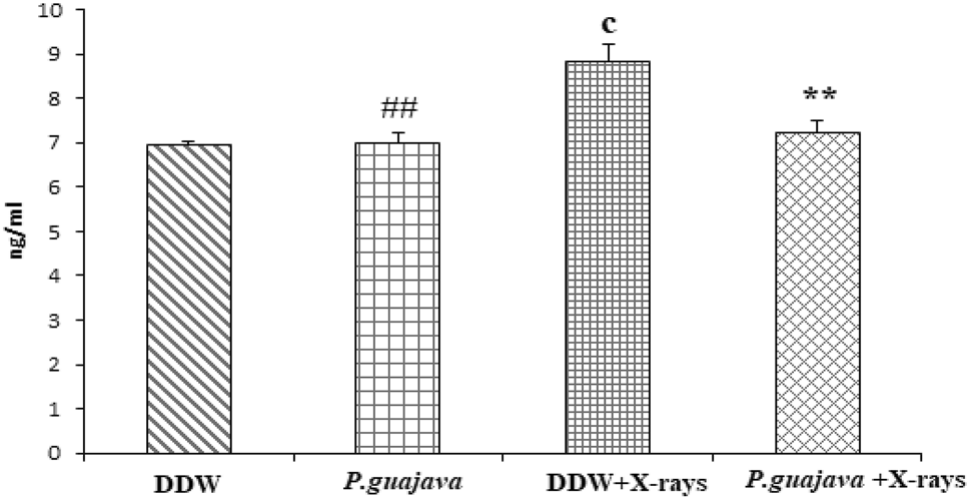
Changes in rat liver COX-2 levels after exposure to X-rays (4 Gy) with or without 200 mg/kg body weight *P. guajava* leaf extracts given orally for five consecutive days. Results are presented as mean ± SEM (*n* = 6). The significance level **, *P* ≤ 0.01 when compared with DDW + X–rays. Whereas the significance levels c, *P* ≤ 0.001 when compared with DDW. The significance levels # #, *P* ≤ 0.01 when compared with DDW + X–rays (4 Gy); analyzed by one-way ANOVA followed by post hoc Tukey test

The animals exposed to a sublethal dose of X-rays (4 Gy) displayed a significant increase in cyclooxygenase -2 levels when compared to animals treated with distilled water (*P* ≤ 0.001). There was no significant difference observed between animals treated with distilled water and *P. guajava* group. The animals pre-treated with 200 mg/kg bodyweight of *P. guajava* prior to X-ray irradiation displayed significant reduction in hepatic cyclooxygenase—2 levels when compared to DDW + X-rays (*P* ≤ 0.01) (Fig. 6). The animals exposed to sublethal dose of X-rays displayed a significant increase in cyclooxygenase -2 levels when compared to *P. guajava* group (*P* ≤ 0.01).

### Estimation of IL-6 in liver tissue

**Fig. 7:**
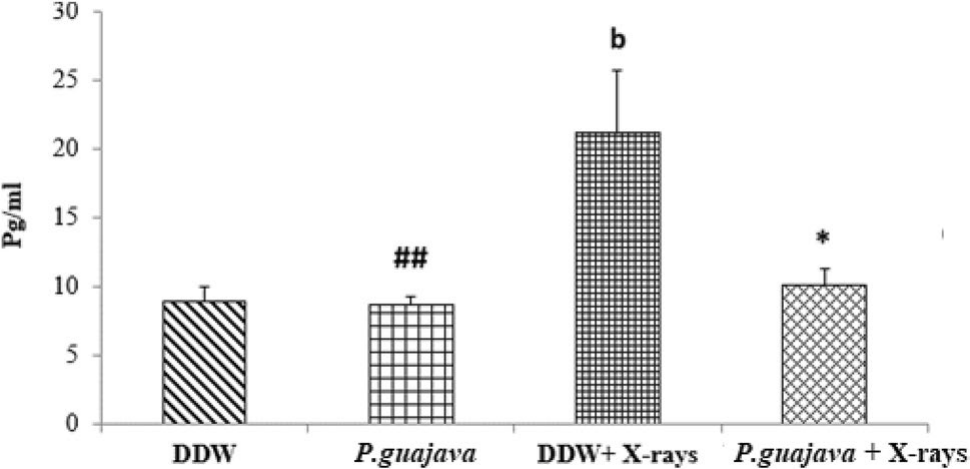
Changes in rat liver IL-6 levels after exposure to X-rays (4 Gy) with or without 200 mg/kg body weight *P. guajava* leaf extracts given orally for five consecutive days. Results are presented as mean ± SEM, (*n*) = 06. The significance levels *, *P* ≤ 0.05 when compared with DDW + X–rays (4 Gy). Whereas the significance levels b, *P* ≤ 0.01 when compared with DDW. The significance levels # #, *P* ≤ 0.01 when compared with DDW + X–rays (4 Gy); analyzed by one-way ANOVA followed by post hoc Tukey test

IL-6 is an endogenous cytokine that is active in inflammation. The levels of interleukin—6 are presented in Fig. 7. The animals exposed to a sublethal dose of X-rays (4 Gy) exhibited a significant increase in interleukin – 6 levels when compared to the distilled water treated group (*P* ≤ 0.01). There was no significant difference observed between groups treated *by P.guajava* and distilled water. Pre-treatment of extract (200 mg/kg body weight) prior to X-ray irradiation displayed significant decrease in hepatic interlukin-6 levels (*P* ≤ 0.05). Significant difference was observed between group treated with *P.guajava* and DDW + X-rays (*P* ≤ 0.01).

### Estimation of IL-10 in liver tissue

**Fig. 8:**
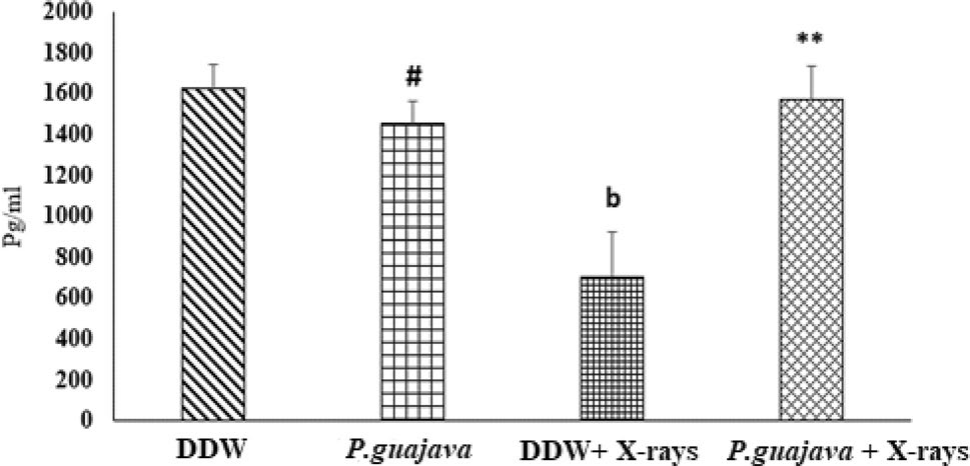
Changes in rat liver IL-10 levels after exposure to X-rays (4 Gy) with or without 200 mg/kg body weight *P. guajava* leaf extracts given orally for five consecutive days. Results are presented as mean ± SEM, (*n*) = 06. The significance level **, *P* ≤ 0.01 when compared with DDW + X–rays (4 Gy). Whereas the significance level b, *P* ≤ 0.01 when compared with DDW. The significance levels #, *P* ≤ 0.05 when compared with DDW + X – rays (4 Gy); analyzed by one-way ANOVA followed by post hoc Tukey test

In the present study, animals exposed to X-rays (4 Gy) displayed a significant reduction in interleukin -10 levels (Fig. 8) when compared with distilled water group (*P* ≤ 0.01). Pre-treatment of 200 mg/kg body weight *P.guajava* prior to X-rays irradiation displayed a significant increase in interleukin -10 levels when compared to DDW + X-rays (*P* ≤ 0.01). No statistical difference was observed between distilled water and *P.guajava* group. The significant difference was observed between group treated with *P guajava* and DDW + X-rays (*P* ≤ 0.05).

### Biochemical estimation in RBC lysate

**Fig. 9:**
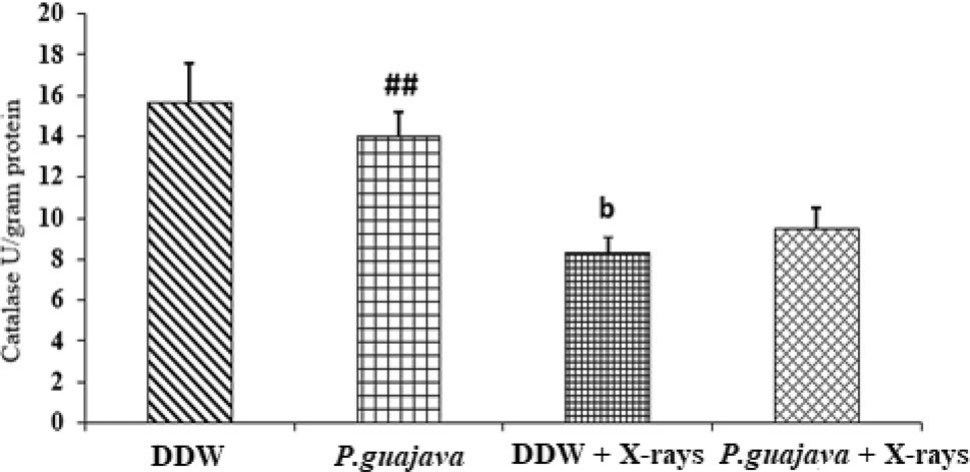
Changes in the activities of catalase in DDW, *P.guajava* (200 mg/kg body weight), DDW + X-rays (4 Gy), *P.guajava* + X-rays (4 Gy) treated rat RBC lysate groups. Results are presented as mean ± SEM (n = 6). DDW + X – rays (4 Gy) vs *P.guajava* + X – rays (*P* = 0.848). The significance levels b, *P* ≤ 0.01 when compared with DDW. Whereas the significance levels # #, *P* ≤ 0.01 when compared with DDW + X – rays (4 Gy); analyzed by one-way ANOVA followed by post hoc Tukey test

**Fig. 10:**
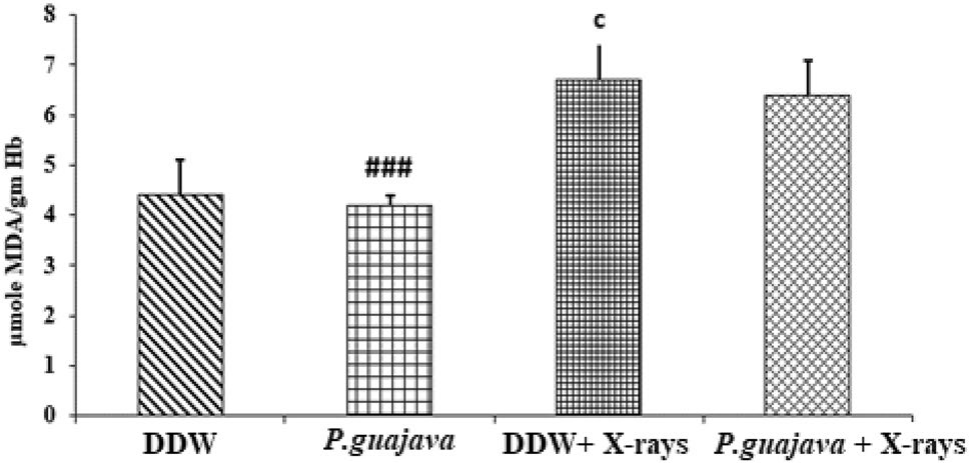
Changes in the activities of lipid peroxidation in DDW, *P.guajava* (200 mg/kg body weight), DDW + X-rays (4 Gy), *P.guajava* + X-rays (4 Gy) treated rat RBC lysate groups. Results are presented as mean ± SEM (*n *= 6). DDW + X-rays (4 Gy) vs *P. guajava* + X-rays (*P* = 0.401). The significance level c, *P* ≤ 0.001 when compared with DDW. Whereas the significance level # # #, *P* ≤ 0.001 when compared with DDW + X-rays (4 Gy); analyzed by one-way ANOVA followed by post hoc Tukey test

**Fig. 11:**
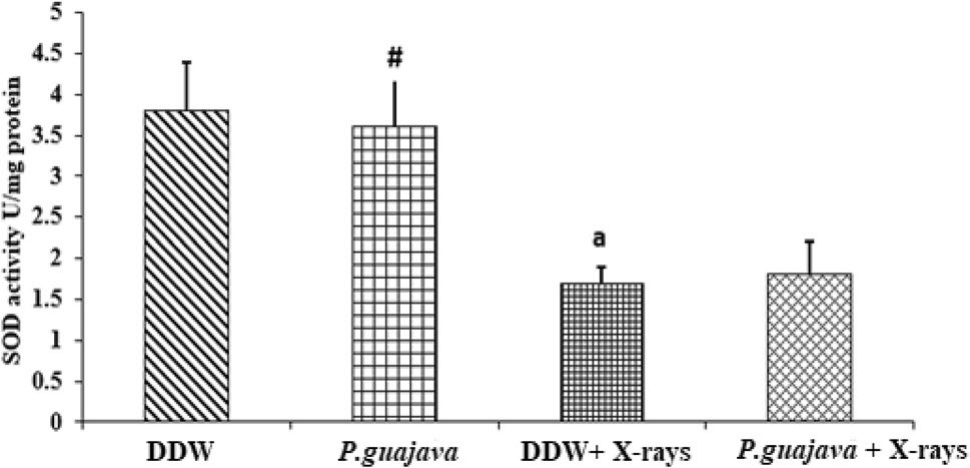
Changes in the activities of superoxide dismutase in DDW, *P. guajava* (200 mg/kg body weight), DDW + X-rays (4 Gy), *P.guajava* + X-rays (4 Gy) treated rat RBC lysate groups. Results are presented as mean ± SEM (*n* = 6). DDW + X-rays vs *P.guajava* + X-rays (*P* = 0.536). The significance levels a, *P* ≤ 0.05 when compared with DDW. Whereas the significance levels #, *P* ≤ 0.05 when compared with DDW + X-rays (4 Gy); analyzed by one-way ANOVA followed by post hoc Tukey test

**Fig. 12:**
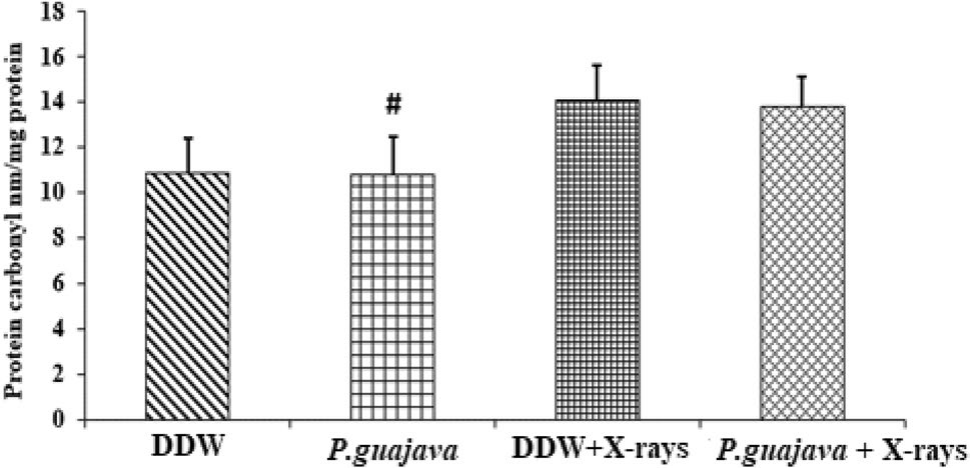
Changes in the activities of protein carbonyl in DDW, *P. guajava* (200 mg/kg body weight), DDW + X-rays (4 Gy), *P. guajava* + X-rays (4 Gy) treated rat RBC lysate groups. Results are presented as mean ± SEM (*n* = 6). DDW + X-rays(4 Gy) vs *P. guajava* + X-rays(4 Gy) (*P* = 0.999). DDW vs DDW + X-rays (4 Gy) (*P* = 0.398). The significance level #, *P* ≤ 0.05 when compared with DDW + X-rays (4 Gy); analyzed by one-way ANOVA followed by post hoc Tukey test

The endogenous cellular catalase, superoxide dismutase, lipid peroxidation and protein carbonyl levels were analyzed to study the involvement of these markers in free radicals scavenging in X-rays treated rats. The levels of antioxidant enzyme catalase (*P* ≤ 0.01) and superoxide dismutase (*P* ≤ 0.05) were significantly reduced in animals treated with sublethal dose of X-rays (4 Gy) when compared with distilled water group. There was steady increase in the antioxidant enzymes catalase (Fig. 9) and superoxide dismutase (Fig. 10) levels in RBC lysate in *P. guajava* pre-treated group but was not statistically significant when compared to DDW + X-rays (4 Gy). Rats exposed to 4 Gy of X-rays displayed significant increase (*P* ≤ 0.001) in the levels of TBARS indicating increased rate of lipid peroxidation when compared with animals treated with distilled water. In *P.guajava* pre-treated group, the reduction of TBARS levels (Fig. 11) were observed but was not statistically significant when compared to DDW + X-rays (4 Gy).The animals exposed to sublethal dose of X-rays (4 Gy) displayed an increase in the protein carbonyl levels but was not statistically significant when compared with animals treated with distilled water,demonstrating protein oxidation in cells (Fig. 12). Animals pre-treated with 200 mg/kg body weight *P. guajava* displayed a slight reduction in protein carbonyls levels but was not significant when compared to DDW + X-rays (4 Gy).

**Fig. 13:**
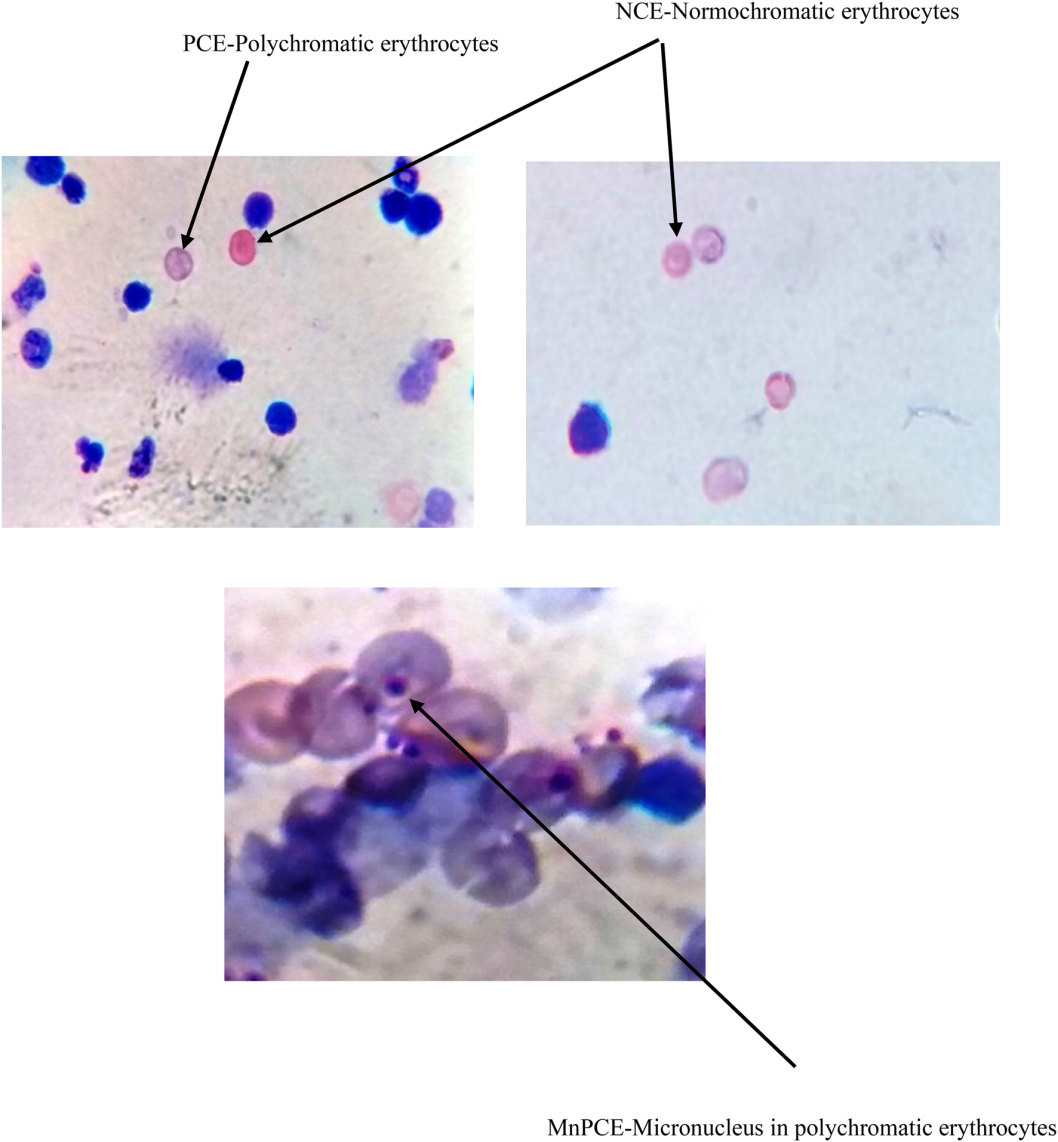
Representative images of micronucleus assay in bone marrow

The effect of sublethal dose of X-rays (4 Gy) irradiation with and without *P.guajava* on the initiation of micronucleus in polychromatic erythrocytes in bone marrow is represented in Table 1. The animals exposed to X-rays (4 Gy) displayed a significant increase (*P* ≤ 0.001) in micronucleus in polychromatic erythrocytes when compared to animals treated with distilled water. Pre-treatment of 200 mg/kg body weight *P.guajava* one hour prior to X-rays irradiation displayed significant reduction (*P* ≤ 0.01) in the generation of micronucleus in polychromatic erythrocytes when compared with DDW + X-rays (Fig. 13). The PCE/NCE ratio increased in *P. guajava* pre-treated group when compared to X-ray irradiated group, respectively.

**Table 1:** Effects of *P. guajava* leaf extract on the formation of radiation-induced micronucleus in polychromatic erythrocytes in rat bone marrow exposed to 4 Gy X-rays

Groups	DDW	*P. guajava*	DDW + X–rays	*P. guajava* + X-rays
PCE	46.48 ± 5.08	48.32 + 4.3	28.64 ± 3.3	36.4 ± 2.5
MnPCE	0	0^###^	16.7 ± 3.9^c^	10.5 ± 2.2^**^
NCE	53.51 ± 4.1	51. 67 ± 7.2	46.41 ± 4.2	45.95 ± 3.6
MnNCE	0	0^###^	8.2 ± 1.1^c^	8.2 ± 1.15
PCE/NCE	0.86	0.93	0.61	0.79

Data expressed as average percentage (%) of cells. Micronucleated polychromatic erythrocytes (MnPCE), the significance levels **, *P* ≤ 0.01 when compared with DDW + X –rays (4 Gy). Whereas the significance level c, *P* ≤ 0.001 when compared with DDW. The significance level # # #, *P* ≤ 0.001 when compared with DDW + X – rays (4 Gy)

*PCE *Polychromatic erythrocytes, *MnPCE *Micronucleus in polychromatic erythrocytes, *MnNCE *Micronucleus in Normochromatic erythrocytes, *NCE *Normochromatic erythrocytes

To study the effect of *P.guajava* on peripheral blood, hematological parameters such as total RBC count, total WBC count and total blood platelets were studied. The data is presented in Table 2. The animals exposed to X-rays (4 Gy) displayed significant reduction in the RBC count (*P* ≤ 0.001) when compared to the groups treated with distilled water. Pre-treatment with *P.guajava* one hour prior to X-rays irradiation displayed a significant increase (*P* ≤ 0.001) in total RBC count when compared with DDW + X-rays. A significant reduction in total WBC count (*P* ≤ 0.001) was observed in animals exposed to sublethal dose of X-rays. Pre-treatment of 200 mg/kg bodyweight for 5 days and one hour prior to X-rays irradiation displayed a significant increase (*P* ≤ 0.05) in the total WBC count when compared to the irradiated group alone. The total platelet levels decreased significantly in X-rays irradiated group (*P* ≤ 0.05) when compared to animals treated with distilled water alone. The animals pre-treated with 200 mg/kg body weight *P.guajava* displayed a significant increase (*P* ≤ 0.05) in total platelet count when compared with DDW + X-rays, respectively.

**Table 2:** Effect of *P. guajava* on hematological changes in X-rays irradiated peripheral blood of albino Wistar rat

Groups	RBC × 10^6^ mm^3^	WBC × 10^3^mm^3^	Platelet count10^5^mm^3^
DDW	6.3 ± 0.3	6.05 ± 0.7	5.76 ± 1.9
DDW + X –rays	2.1 ± 2.83^c^	3.47 ± 0.32^c^	3.85 ± 0.4^a^
*P. guajava*	5.91 ± 1.90^# # #^	6.6 ± 0.2^# # #^	6.7 ± 0.23^# #^
*P. guajava* + X – rays	4.4 ± 1.45^***^	5.75 ± 1.19^*^	5.3 ± 1.9^*^

Each value represents Mean ± SEM. Total RBC count ***, *P* ≤ 0.001 when compared with DDW + X –rays (4 Gy) c, *P* ≤ 0.001 when compared with DDW # # #, *P* ≤ 0.001 when compared with DDW + X –rays (4 Gy). WBC count *, *P* ≤ 0.05 when compared with DDW + X –rays (4 Gy) c, *P* ≤ 0.001 when compared with DDW # # #, *P* ≤ 0.001 when compared with DDW + X –rays (4 Gy). Platelet count *, *P* ≤ 0.05 when compared with DDW + X –rays (4 Gy) a, *P* ≤ 0.05 when compared with DDW # #, *P* ≤ 0.01 when compared with DDW + X –rays (4 Gy)

**Fig. 14:**
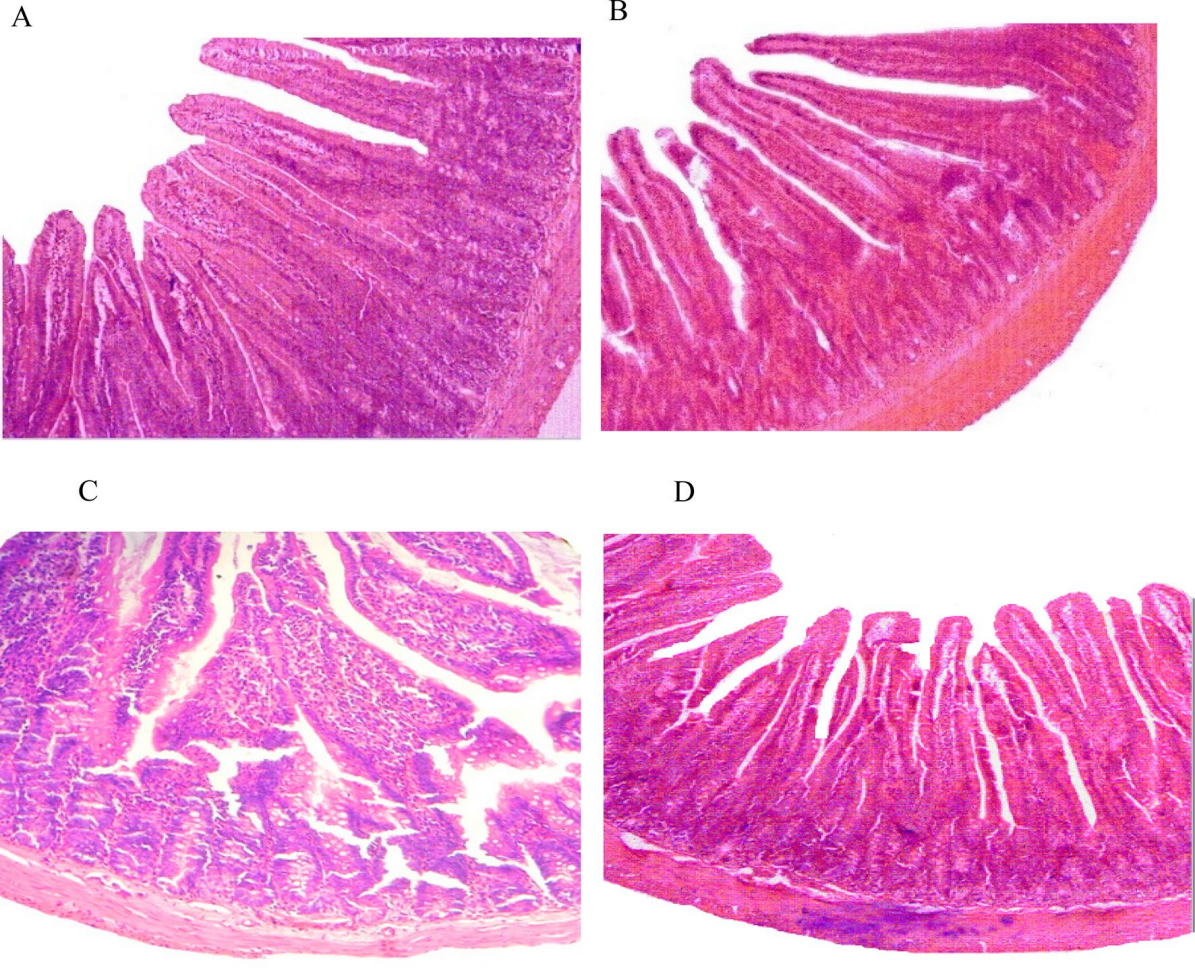
Histopathological demonstration of protective effect of *P.guajava* in the small intestine of irradiated rat. Photomicrograph of jejunum sections stained with H&E staining. **a** DDW, **b P**. *guajava*, **c** DDW + X-rays (4 Gy), **d** *P. guajava* + X-rays (4 Gy); Magnification – 20X

The animals exposed to sublethal dose of X-rays (4 Gy) resulted in the conspicuous modification of small intestine. Gastrointestinal damage is a characteristic hallmark of acute radiation syndrome. In the present study histopathology of small intestine displayed degeneration, blunting of villi and distorted architecture (Fig. c). Pre-treatment of 200 mg/kg bodyweight of *P. guajava* for five consecutive days prior to X-rays irradiation displayed normal architecture (Fig. 14) of intestine thereby offering protection from radiation induced intestinal damage.

## Discussion

**Fig. 15:**
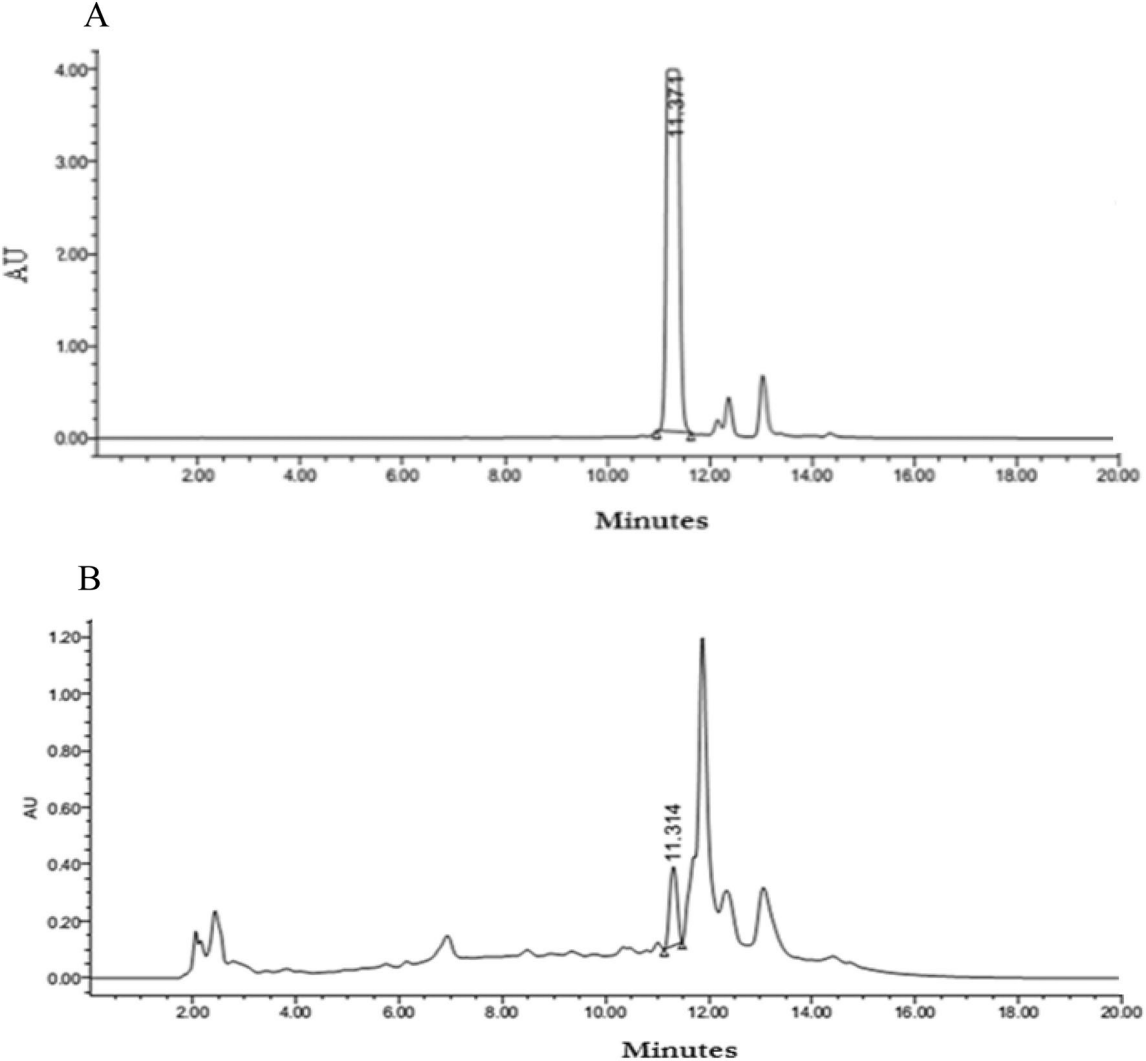
**a** HPLC chromatogram of reference standard quercetin, **b **HPLC chromatogram of *P. guajava* extract

**Fig. 16:**
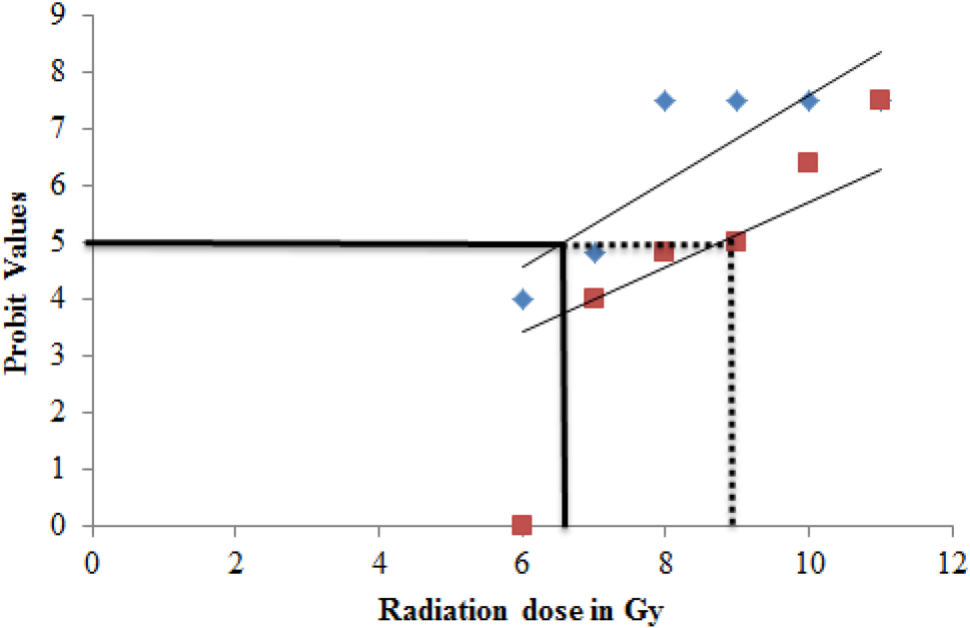
Radiation dose response curve for the 30-day survival of rats after whole-body X-ray irradiation (7 Gy, 8 Gy, 9 Gy, 10 Gy, 11 Gy) in *P. guajava* pretreated group. Radiation dose is represented in log doses. Percentage mortalities are represented as probit values in the Y-axis

It has been documented that when living cells are exposed to lethal radiation, they display numerous changes depending on radiation dose rate, duration of dose and tissue sensitivity Shimura and Kojima (2018). X-rays and other forms of ionizing radiation damage tissue by chain reactions caused by reactive oxygen and nitrogen species (Azzam et al. 2012). There is an unrecoverable depletion of antioxidants in tissues exposed to high doses of ionizing radiation (Okunieff et al. 2008). Although living cells are equipped with endogenous antioxidants that protect cells from damage, an increase in the production of free radicals by ionizing radiation cannot be restricted by endogenous antioxidants only. As a result, the protection offered by these antioxidants during radiation exposure is negligible. The ability to scavenge reactive oxygen and nitrogen species is a requisite in the development of powerful radiation mitigators (Citrin et al. 2010). DPPH scavenging activity is a widely used method to screen free radical scavenging properties of compounds. In the present study, *P.guajava* scavenged DPPH free radicals and nitric oxide in a dose-dependent manner. This is in agreement with the work published by Adesida and Farombi (2012). A concentration of 10 μg/ml displayed 78.80% DPPH scavenging activity (Fig. 4). While nitric oxide is an essential chemical mediator of physiological processes in vivo, it can also generate free radicals in aerobic conditions. Plant products are found to scavenge nitrogen derived free radicals as well (Hunyadi 2019). 10 µg/ml of *P.guajava* leaf extract displayed a nitric oxide scavenging activity of 53.1% (Fig. 5). This activity of *P.guajava* could be attributed to the presence of high quantity of polyphenols. Total phenolic content of 32.5 ± 4.33 mg GAE/g of *P.guajava* extract has been reported by us earlier (Kumar et al. 2015) Moreover, quercetin being a flavonoid is a powerful scavenger of free radicals and is found to be an important component of the leaf extract as depicted by HPLC (Fig. 15). Biochemical estimations in RBC lysates indicated that *P.guajava* did not contribute remarkably to the antioxidant activity in blood, as no significant change was observed with respect to the levels of lipid peroxidation, protein carbonyls, catalase and SOD although its contribution to hepatic antioxidant activity was highly significant. This suggests that the action of phytochemicals of *P. guajava* leaf extract is differential and is restricted to tissues, liver being one of the predominant tissues. Oxidative stress often culminates in inflammation within the tissue, which can be evaluated by the levels of inflammatory markers. In this study, we have evaluated the hepatic levels of COX-2, IL-6 and IL-10. In earlier studies, it has been noted that COX-2 enzyme is overexpressed and involved in radiation induced cellular damage (Steinauer et al. 2000). Ionizing radiations such as X-rays and γ-rays activates cytoplasmic phospholipase A_2_ which produces arachidonic acid from membrane phospholipids causing overproduction of cyclooxygenase-2 enzyme. Prostaglandin E2 is a vital product of cyclooxygenase-2 enzyme that is generated in response to X-rays and other ionizing radiation exposure, which mediates liberation of free radicals and nitric oxide. Upregulation of COX-2 gene expression has been stated by certain studies involving exposure of rats to a wide range of radiation doses (Choy 2003). We report similar effects in our study, where animals exposed to sublethal dose of X-rays (4 Gy) displayed significant increase in COX-2 levels (*P* ≤ 0.001) when compared to animals treated with distilled water. It has been reported in earlier studies that antioxidants significantly decrease prostaglandin production by inactivating cyclooxygenase—2 catalytic activity levels at transcriptional level (Chinery et al. 1998). We report similar effects in our study. Pre-treatment of *P.guajava* prior to X-rays exposure displayed significant reduction (*P* ≤ 0.01) in cyclooxygenase-2 levels when compared to animals treated with DDW + X-rays (Fig. 6). In earlier studies, Chen et al. (2012) reported that there was a significant increase in IL-6 levels in the cellular supernatants and serum in mice exposed to radiation. The proinflammatory cytokine IL-6 levels were significantly elevated in patients undergoing radiotherapy when analyzed in serum (Kiprian et al. 2018). In the present study, we observed similar results, where animals exposed to radiation (4 Gy) displayed a significant increase (*P* ≤ 0.01) in interleukin-6 levels when compared to animals treated with distilled water. In earlier studies, Korish (2010) reported that supplementation of antioxidants to rats significantly reduced interleukin-6 levels and subsequently reduced chronic renal failure. Pre-treatment with *P. guajava* before X-rays irradiation displayed significant reduction (*P* ≤ 0.05) in interleukin-6 levels when compared to the irradiated group (Fig. 7) indicating that *P.guajava* is a potent source of antioxidants (Camarena et al. 2018). The anti-inflammatory cytokine interleukin-10 with its cell mediated immune response and anti-inflammatory action has vast potential in treating inflammatory, autoimmune disorders and rheumatoid arthritis. In earlier studies, Li et al. (2016) reported that plasma level of IL-10 was significantly reduced in subjects who underwent radiation therapy. The animals exposed to 4 Gy X-rays displayed a significant reduction in hepatic interleukin-10 levels when compared to animals treated with distilled water (*P* ≤ 0.01). Pre-treatment of *P.guajava* (200 mg/kg body weight) displayed a significant increase in the anti-inflammatory marker, interleukin 10 levels when compared to the irradiated group (Fig. 8). Thus in the present study, we observed that the anti-inflammatory marker interleukin-10 has decreased and pro-inflammatory markers like cyclooxygenase 2 and interleukin-6 levels have increased suggesting inflammation in hepatic tissue in animals exposed to radiation. Micronucleus is considered as an appropriate biomarker to study the clastogenic hazard. In the earlier study, Nair et al. (2013) reported that mice exposed to ionizing radiation (2 Gy) displayed a significant increase in micronucleus in polychromatic erythrocytes when compared to normal control group. Similar observations were reported by Boroujeni et al*.* where mice exposed to 2 Gy gamma radiation displayed significant rise in micronucleus in polychromatic erythrocytes at 24 and 48 h after radiation exposure (Boroujeni et al. 2016). In the present study, similar findings were observed, as animals exposed to sublethal dose of X-rays (4 Gy) displayed significant increase (*P* ≤ 0.001) in the micronucleus in polychromatic erythrocytes (Fig. 13) when compared to animals treated with distilled water. The polyphenolic compounds present in plants have antioxidant properties and protect genetic material from free radicals such as hydroxyl radicals produced by ionizing radiation. Flavonoids and polyphenols present in plants displayed a significant reduction in genotoxicity in mice exposed to ionizing radiation (Koohian et al. 2017). Pre-treatment with *P. guajava* one hour prior to X-rays displayed a significant reduction in micronucleus in polychromatic erythrocytes (*P* ≤ 0.01) when compared to irradiated group (Table 1). Blue–green algae and certain plants like *P. niruri*, are also reported to protect mice bone marrow against radiation induced micronucleus formation (Thakur et al. 2011). Abundant proliferating cells are present in the intestinal epithelium making it highly sensitive to ionising radiation. Earlier studies reported that mice exposed to ionizing radiation (6 Gy) displayed alteration in tissue ultra-structure in intestinal epithelium and also resulted in death of the crypt cells (Guruvayoorappan and Kuttan 2008). Similar observations were documented in the present study in animals exposed to a sublethal dose of X-rays (4 Gy). The animals pretreated with *P. guajava* regenerated the regular architecture of the small intestine (Fig. 14). Animals irradiated with 4 Gy X-rays resulted in a significant decline in RBC, WBC and platelet count due to the damage caused to bone marrow. Similar observation was reported in BALB/c mice exposed to 5 Gy radiation (Huang et al. 2016). Pre-treatment with 200 mg/kg body weight *P. guajava* for five consecutive days prior to X-ray irradiation prevented significant decline in RBC, WBC and platelet counts bringing back the values to near normal suggesting the role of *P. guajava* as an immunostimulant. Similarly, plant preparation such as *Tragia involucrate* and *Saraca indica* have been reported to improve the hematological parameters in mice exposed to whole-body radiation (Thimmaiah et al. 2019; Rao et al. 2010). Preliminary HPLC analysis of *P.guajava* indicated the presence of quercetin (Fig. 15) in the hydroalcoholic leaf extract. Quercetin has proved its beneficial role as radioprotector by reducing genotoxicity in mice exposed to ionizing radiation of 4 Gy (Benkovic et al. 2009). Quercetin-3-*O*-rutinoside, a flavonoid restored intestinal damage, reduced apoptosis in splenocytes and reduced genotoxicity significantly in mice exposed to whole-body γ-radiation (Bansal et al. 2012). Flavonoids such as quercetin and rutin play a vital role in ameliorating radiation induced damage. The animals exposed to 7 Gy, 8 Gy, 9 Gy, 10 Gy and 11 Gy of X-rays displayed 0%, 0%, 50%, 83.33% and 100% mortality, respectively. The animals exhibited behavioural changes, intense diarrhea, irritability, and erythema in 2 to 3 days of whole-body X-ray exposure. In our previous study we found that LD_50/30_ of X-ray was found to be 6.6 Gy (Srinivas et al. 2015). In the present study, pre-treatment of *P. guajava* before irradiation reduced the severity of acute radiation syndrome in a concentration dependent manner. The survival of animals was solely due to the protection rendered by *P. guajava* to the differentiating cells present in the gastrointestinal tract and bone marrow. The animals administered with 200 mg/kg body weight *P. guajava* for five consecutive days before X-rays irradiation increased the mean lethal dose up to 9 Gy. Finally, any plant extract can be authenticated for its role as a radioprotector by calculating its DRF, which was found to be 1.36 for *P. guajava* (Fig. 16). This DRF may not be that of an ideal radioprotector. Perhaps a repetition of biochemical, histological and clastogenic effects using the lead molecule from the leaf extracts of *P. guajava* may yield valuable information on radioprotection.

## Conclusion

Our research conducted so far suggests that *P.guajava* at dose of 200 mg/kg body weight demonstrated protection against radiation induced damages and served as a radioprotector in animal model. The same results may be extrapolated in humans as well. Furthermore, the compound responsible for the protective effect needs to be isolated and repeated in human clinical trials with special reference to patients undergoing radiotherapy.
